# Supplementary material for: Pharmacist-Driven Antibiotic Stewardship Program in Febrile Neutropenic Patients: A Single Site Prospective Study in Thailand
Source: Antibiotics (Basel). 2021 Apr 17;10(4):456. doi: 10.3390/antibiotics10040456 (PMC8072986; doi:10.3390/antibiotics10040456)
Supplement: Supplementary file 1 [file antibiotics-10-00456-s001.zip › antibiotics-1174004-supplementary.pdf]

Supplementary data

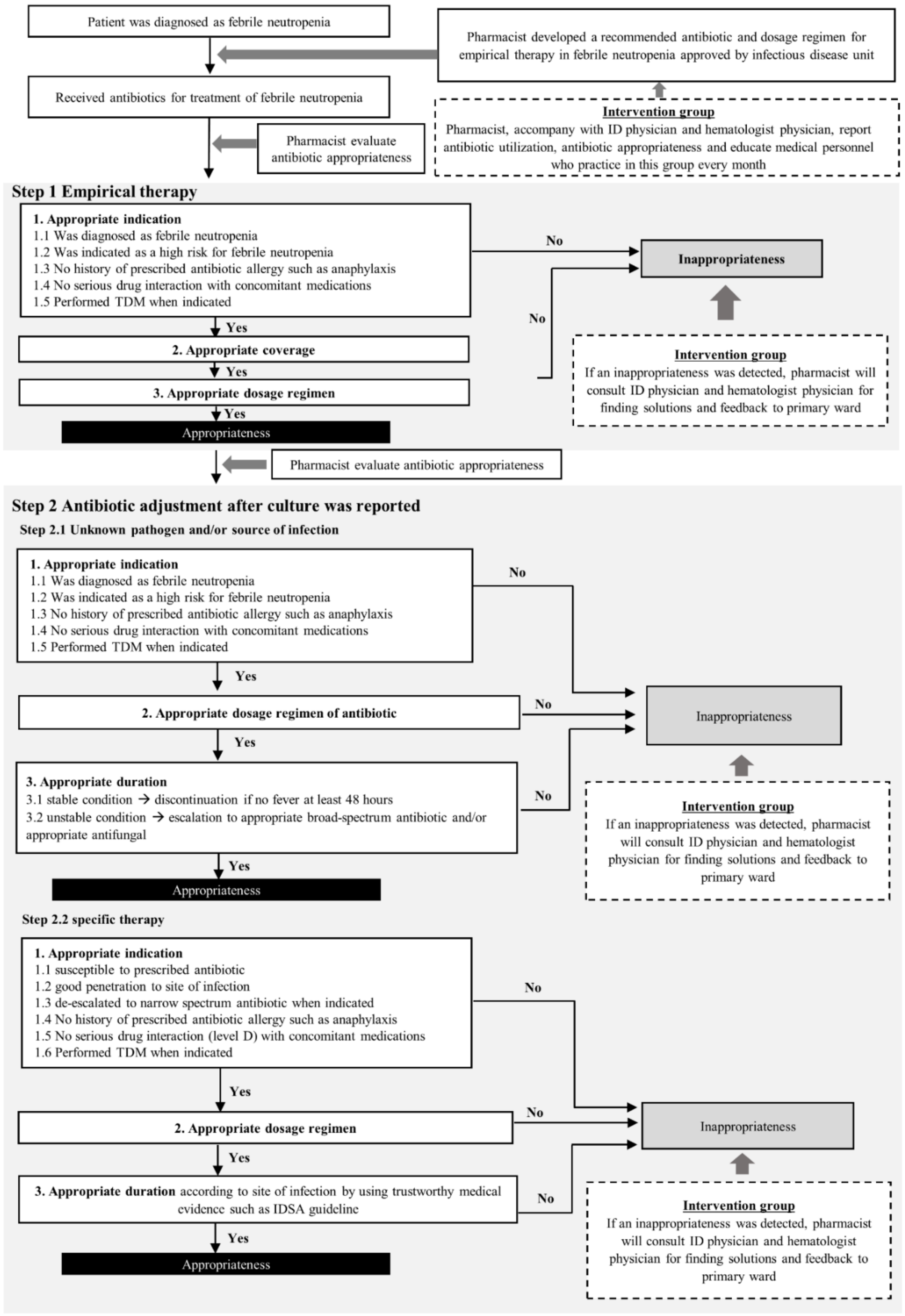

Figure S1. Criteria of antibiotic appropriateness evaluation
ID, infectious diseases; IDSA, Infectious Disease Society of America; TDM, Therapeutic drug monitoring
Therapeutic drug monitoring was referred to vancomycin and voriconazole monitoring.
Stable condition was defined as no hemodynamic instability condition, no fever at least 48 hours.

Table S1. Antibiotics utilization

DDD/1000 patient day	Intervention group				Control group			
	Mean ± SD	coefficient	Standard error	*P* value	Mean ± SD	coefficient	Standard error	*P* value
Ceftazidime	206.56 ± 176.20	-4.286	24.264	0.865	211.04 ± 128.47	-22.390	15.581	0.194
Cefepime	53.79 ± 69.55	-0.343	9.598	0.972	42.30 ± 38.68	5.927	27.268	0.656
Piperacillin/tazobactam	388.60 ± 163.01	20.315	21.147	0.369	288.75 ± 131.69	1.915	18.161	0.919
Meropenem	194.43 ± 88.19	-6.363	11.932	0.610	163.01 ± 79.39	17.472	8.743	0.086
Imipenem	38.61 ± 81.75	3.801	11.191	0.744	0.00	-	-	-
Total target antibiotics	882.00 ± 280.30	13.124	38.366	0.742	705.10 ± 193.01	2.923	26.615	0.916
Total antibiotics	1249.32 ± 475.86	-2.726	65.666	0.968	1113.63 ± 242.63	48.308	27.975	0.128

DDD, daily defined dose; SD, standard deviation

Table S2. Antibiotic appropriateness in documentation therapy: unknown source of infection

Outcomes	Intervention (20 FN episodes), No. (%)	Control (22 FN episodes), No. (%)	*P* value
Overall appropriateness	18 (40)	12 (26.7)	0.039
Appropriate indication	20 (44.4)	22 (48.9)	0.416
Appropriate dosage regimen	20 (44.4)	20 (44.4)	0.638
Appropriate duration	18 (40)	13 (28.9)	0.066

FN, febrile neutropenic

Table S3. Antibiotic appropriateness in documentation therapy: known causative pathogens and source of infection

Outcomes	Intervention (24 FN episodes), No. (%)	Control (23 FN episodes), No. (%)	*P* value
Overall appropriateness	23 (51.1)	17 (37.8)	0.116
Appropriate indication	23 (51.1)	19 (42.2)	0.394
Appropriate dosage regimen	24 (53.3)	22 (48.9)	0.833
Appropriate duration	23 (51.1)	21 (46.7)	0.872

FN, febrile neutropenic
